# The Hospital World

**Published:** 1911-03-04

**Authors:** 


### The Hospital World.

A successful business and philanthropic career with a genuine dash of romance about it came to an end with the death of Mr. William Bradbury Robinson, at the age of eighty-four, last week. The best summary of this we have seen appeared in the *Times*: "Mr. William Bradbury Robinson was one of the best-known public men in Derbyshire, and one of the pioneers in the country of medical lint manufacture. He was practically the founder and the head of the firm of Robinson & Sons (Limited), the patentees and manufacturers of medicated lint, surgical bandages, tissue, etc. The son of a chemist, seeing the need of a satisfactory article he set himself to solve the rapid manufacture of lint and surgical bandages. In 1854 he came across a small machine which had been worked by a woman in the county town, and bought it and any rights possessed in it for 27s. 6d. Mr. Robinson, by means of mechanical improvements, was able to produce a machine which was capable of putting on the market a cheaper and a better article. From that small beginning a great manufactory employing many hundreds of hands has been built up. From the weaving of cloth followed the lint industry, bleaching followed the calico weaving, then came the manufacture of absorbent cotton wool, antiseptic dressings, and medicated bandages. Enormous consignments were used in the army hospitals during the Russo-Japanese War, and it was the proud boast of Mr. Robinson that during the South African war his firm sent enough cottonwool to South Africa to wrap up the whole British Army in! The firm is contractor to most of the London hospitals."

